# Nurses' Experience of Redeployment to a New Intermediate Care Unit for Respiratory Patients: A Qualitative Study

**DOI:** 10.1111/jan.70170

**Published:** 2025-08-22

**Authors:** Francesca Marangon, Michela Bottega, Stefania Avoni, Mayra Veronese, Matteo Danielis

**Affiliations:** ^1^ Pulmonology Unit Azienda Unità Locale Socio‐Sanitaria (AULSS) 2 Marca Trevigiana Treviso Italy; ^2^ Chief Nurse Office, Department of the Health Care Professions Azienda Unità Locale Socio Sanitaria (AULSS) 2 Marca Trevigiana Treviso Italy; ^3^ Laboratory of Studies & Evidence‐Based Nursing, Department of Cardiac, Thoracic, Vascular Sciences and Public Health University of Padova Padova Italy

**Keywords:** change management, critical care, nurses' experiences, qualitative research, redeployment

## Abstract

**Aim:**

To explore the experiences of nurses transitioning from a clinical ward to a newly established respiratory intermediate care unit (IMCU).

**Design:**

A qualitative descriptive approach was adopted to capture the lived experiences of redeployed nurses. This design was selected to address the research question: What are the initial experiences of nurses transitioning from general ward settings to a newly established IMCU for respiratory patients?

**Methods:**

Two focus groups were conducted in June 2024, involving 14 purposefully selected registered nurses. Data were analysed using Braun and Clarke's thematic analysis framework, with the study reported in line with the Standards for Reporting Qualitative Research.

**Results:**

The analysis revealed two interconnected themes reflecting the complexity of the redeployment experience. The first theme, ‘The introspection of waiting amidst change and readiness’, captures the emotional ambivalence nurses felt, characterised by anticipation, uncertainty and a perceived lack of preparedness. This phase was marked by concerns over clinical competence, fear of errors and the weight of new legal and ethical responsibilities. The second theme, ‘The road to organizational change with both driving forces and obstacles’, highlights nurses' concerns about physician readiness, feeling undervalued and limited involvement in planning. At the same time, nurses emphasised the importance of teamwork, structured preparation, experiential training and having the right equipment.

**Conclusion:**

The study underscores the complexity of role transitions for nurses moving into semi‐critical care settings like IMCUs. It reveals the need for targeted support strategies to reduce uncertainty and enhance role readiness.

**Implications for the Profession and Patient Care:**

To improve the redeployment experience and patient outcomes, healthcare organisations should prioritise structured training, tailored preceptorship programmes and inclusive decision‐making processes. These measures can strengthen nurses' resilience, support workforce sustainability and ensure the delivery of high‐quality, patient‐centred care in intermediate care environments.

**Impact:**

This study highlights the significant impact of inadequate preparation and communication on redeployed nurses' experiences in respiratory IMCUs, emphasising the need for structured training and supportive team dynamics. These findings can guide healthcare leaders, nurse managers and policymakers in developing evidence‐based redeployment strategies that reduce anxiety, strengthen team cohesion and ultimately improve nurse adaptation and patient care in semi‐critical settings.

**Reporting Method:**

We used the SRQR guidelines for reporting qualitative studies.

**Patient or Public Contribution:**

No patient or public contribution.

**Summary**

- What does this paper contribute to the wider global clinical community?
  ○ Challenges in redeploying nurses to respiratory intermediate care units (IMCUs), including lack of preparation, role‐related anxiety and organisational barriers.
  ○ Redeployed nurses reported anxiety linked to inadequate preparation, communication gaps and limited decision‐making involvement. However, strong team cohesion and structured training were identified as critical supports.
  ○ Healthcare leaders, nurse managers and policymakers can use these insights to design better redeployment strategies, enhancing nurse adaptation and patient care in semi‐critical settings.

## 1 Introduction

The proper allocation of nursing resources remains a significant challenge in healthcare settings (Tamata and Mohammadnezhad 2023). The introduction of intermediate care units (IMCUs), while not entirely new on a global scale (Plate et al. 2017), represents an emerging organisational model in certain regions. Their implementation adds layers of complexity to resource management and staff distribution across hospital departments, especially in areas where such units have only recently been introduced or are still developing (Sibilio et al. 2024).

IMCUs effectively alleviate strain on intensive care units (ICUs) while providing care for semi‐critical patients. Functioning as ‘step up’ or ‘step down’ units, IMCUs bridge the gap between general wards and ICUs, thereby optimising patient flow and resource use (Armstrong et al. 2015; Plate et al. 2017). The Coronavirus disease 2019 (COVID‐19) pandemic required the rapid reconfiguration of hospital units into temporary IMCUs to accommodate the escalating demands for critical care services (Arabi et al. 2021). Even as the pandemic has subsided, IMCUs have solidified their role as a crucial component of healthcare systems. This intermediate care setting optimises resource allocation, enhances surge capacity, ensures continuity of care and has demonstrated cost‐effectiveness (López‐Jardón et al. 2024). With their ability to adapt staffing and infrastructure, IMCUs have emerged as a sustainable solution for delivering high‐quality care across the acuity spectrum, making them integral to the post‐pandemic healthcare landscape.

Nurse redeployment during the COVID‐19 crisis was widespread and heterogeneous. Although many nurses reported professional growth and the acquisition of new skills (Danielis et al. 2021; Fagerdahl et al. 2022), others experienced moral distress, disruptions in care continuity and a lack of structured support (Fagerdahl et al. 2022). These challenges had significant and concerning implications for nurse well‐being, performance and staff retention (Hartley et al. 2025). Despite these difficulties, the practice of staff reassignment is likely to remain integral to achieving patient safety within health service delivery—especially in light of the ongoing workforce shortage and the shift toward more flexible work arrangements (Abdulmohdi and Huang 2025; Hartley et al. 2024).

## 2 Background

In the nursing literature, work redeployment is understood as a movement from one state or role to another, often triggered by change and characterised by evolving phases (Arrowsmith et al. 2016). Transitions can be linear or complex, shaped by individual, relational and organisational factors (Arrowsmith et al. 2016). In addition, Dunn et al. (2020) identified three possible approaches to redeployment: voluntary, lottery‐based and mandatory redeployment. Moving into unfamiliar settings—particularly in critical care areas—can evoke feelings of vulnerability, perceived deskilling and reluctance to express uncertainties, especially in the absence of tailored support. These factors can heighten stress and negatively impact retention rates (Abdulmohdi and Huang 2025; Gill and Shanta 2020; Windey and McGuire 2020). A qualitative study by Lal et al. (2025) explored the experiences of 164 healthcare workers in the UK during COVID‐19 redeployments. The study highlighted that a lack of autonomy and inadequate communication in redeployment decisions led to feelings of disempowerment and increased stress among staff. Conversely, when healthcare workers were involved in redeployment decisions, they reported a greater sense of control and job satisfaction (Lal et al. 2025). The literature supports the implementation of strategies such as structured orientation, education and training programmes, clinical mentorship, formal preceptorship and team‐based nursing approaches to promote successful transitions (Gohery and Meaney 2013; Lauck et al. 2022; Luk et al. 2025).

Despite the rapid proliferation of IMCUs during the COVID‐19 pandemic and the urgent staff redeployment at that time, routine nurse transfers to new care units have now become a standard practice as part of the healthcare system's redesign to address nursing shortages and patient needs. However, research exploring the experiences of those involved in this process remains limited. IMCUs are intended to treat a wide variety of patients who do not require ICU level care but who do need more frequent nursing care, specialised therapies, and/or higher levels of monitoring than can be given on a general ward (Plate et al. 2017; Sibilio et al. 2024). These characteristics set the IMCU experience apart from other types of nurse redeployment.

## 3 The Study

This study aims to investigate the initial experiences of nurses transitioning from general ward settings to a newly established IMCU for respiratory patients. Understanding these experiences is essential for designing supportive strategies that align with evolving care models, foster retention, and uphold high standards of patient safety and quality. The findings will offer valuable insights to nurse managers and professional development specialists, enabling them to provide targeted support that optimises redeployment, promotes resilience, and improves the overall delivery of healthcare. The study is guided by the following research question: What are the initial experiences of nurses transitioning from general ward settings to a newly established IMCU for respiratory patients?

## 4 Methods

### 4.1 Design

A qualitative descriptive study was conducted, drawing on the interpretive description methodology developed by Thorne et al. (2004). Rooted in a constructivist and naturalistic paradigm, this approach is particularly well‐suited for nursing research by emphasising practical implications (Hunt 2009; Thorne et al. 2004).

It acknowledges researchers' pre‐existing clinical and theoretical expertise as valuable for generating meaningful insights into complex healthcare phenomena, supporting applied understanding relevant to practice and policy (Hunt 2009). This design aligns with the study's aim to inform nursing leadership and workforce development. Reporting follows the Standards for Reporting Qualitative Research (SRQR) (Table S1) (O'Brien et al. 2014).

### 4.2 Study Setting and Sampling

The study was conducted in a 1000‐bed tertiary hospital in northeastern Italy. During the COVID‐19 emergency, the respiratory ward was mandatorily repurposed, with 20 beds converted to intermediate care beds for patients requiring low‐ to medium‐intensity respiratory support. This ward was later relocated to a newly established ward within the same hospital, which now comprises 33 beds: 26 allocated to stable respiratory patients and seven designated for the new respiratory IMCU. The staff of the new unit includes registered nurses (RNs), medical doctors (MDs), nurse assistants (NAs), physiotherapists and respiratory therapists. The nurse‐to‐patient ratio is 1:4.

Purposeful sampling was employed to recruit participants most informative for the study (Palinkas et al. 2015). Inclusion criteria were: (1) registered nurses (RNs) with at least 1 year of experience in the respiratory clinical setting (Chicca and Bindon 2019; Lartey et al. 2014; Schmitt and Schiffman 2019); (2) direct involvement in the transition from a general respiratory ward to the newly established IMCU; and (3) willingness to participate in the study. Of the 20 RNs eligible for inclusion—defined as those who had been redeployed from general ward settings to the newly established IMCU for respiratory patients—all were invited via a standardised email from the principal investigator. The email outlined the study's purpose, procedures and voluntary nature. To ensure transparency and minimise recruitment bias, no follow‐up emails were sent and all responses were documented. Six nurses declined participation due to personal or professional obligations, resulting in a final sample of 14 RNs.

### 4.3 Data Collection

Data were collected through two focus groups in June 2024, each including seven RNs. Focus groups encourage open discussion by fostering a supportive environment where participants can share and reflect on experiences, often generating richer data than individual interviews (Doyle et al. 2020; Krueger and Casey 2014; Tracy 2019).

The focus groups were conducted by four researchers with diverse professional backgrounds. The moderator, M.D. (male), was an Assistant Professor in Nursing with a PhD and experience in qualitative research. The first recorder, M.B. (female), was a Nurse Manager at the hospital where the study was conducted, also with a PhD and qualitative research experience. The second recorder, S.A. (female), was a Nurse Educator with a Master of Science in Nursing and Midwifery and prior experience in nursing education. The observer, F.M. (female), was a Master of Science in Nursing and Midwifery student with 3 years of clinical experience as an RN. Among the research team, only one member—the student—had prior relationships with some of the participants, having previously worked alongside them during a clinical placement in the same ward. In this study, the student took on the role of a non‐participant observer and did not conduct the interviews. The familiarity was limited to a professional context and did not involve personal relationships. The rest of the research team had no prior contact with the participants.

An interview guide, which served to support discussion without being shared with participants in advance (Table S2), included open‐ended questions, probing questions and prompts, structured around six key dimensions. These dimensions were aligned with the research questions, specifically the important factors influencing the transition process according to the model of Schumacher and Meleis (1994): meanings, expectations, level of knowledge/skill, environment, level of planning and emotional/physical well‐being. A brief sociodemographic data collection form was administered, capturing information on gender (female, male), age (in years), level of education (nursing diploma, Bachelor of Science in Nursing, advanced education), overall working experience (in years) and specific working experience in the adult respiratory unit (in years).

Each session began with a visual stimulus showing a seven‐bed IMCU. Focus groups were audio recorded in a quiet, private hospital meeting room arranged to facilitate open communication, lasting approximately 60 min.

Researchers maintained a non‐judgmental stance, encouraging free expression. At session end, a small buffet and token of appreciation were offered to foster rapport and allow participants to voice any concerns. After each focus group, the team debriefed to share observations and reflect on the process (Krueger and Casey 2014). Recordings were transcribed verbatim by the first author. All researchers reviewed the transcripts to identify emerging insights, which informed the ongoing data collection and analysis (Hunt 2009; Thorne et al. 2004).

Data collection continued until thematic saturation was reached—defined as the point at which no new themes or substantial insights emerged from successive interviews (Vasileiou et al. 2018), as judged by the researchers. Saturation was achieved after the 10th interview; however, all 14 interviews were conducted, as the full sample was available and willing to participate. The final four interviews served to confirm the stability and completeness of the thematic findings.

To ensure the respect of anonymity, quotes were indexed as being from one of the focus groups (e.g., FG1), and assigned a sequentially numbered for each RN (e.g., RN1).

### 4.4 Data Analysis

We conducted a thematic analysis following Braun and Clarke's (2006) six‐phase framework. The process began with data familiarisation, during which researchers read and re‐read transcripts to immerse themselves in the content. The researchers took notes on potential recurring ideas and started thinking about possible codes. Three researchers then independently generated initial codes, identifying and labelling meaningful patterns through the software ATLAS.ti version 22. At this stage, the researchers' focus was on what participants said. It was important to also provide code definitions, as they helped the researchers make decisions about whether to assign this code or another one to a segment of data. These initial codes were collaboratively reviewed and agreed upon. Searching for themes was the third step; the researchers grouped codes into potential themes through team discussion and iterative comparison. Themes were assessed for internal coherence and clear distinction from one another. This process included checking to see if the themes fit in relation to the coded excerpts and if the themes fit in relation to the whole data set. Final themes were named and defined to capture their core meaning, both individually and in relation to one another. Themes were developed inductively, incorporating both within‐ and across‐group patterns (Hunt 2009; Thorne et al. 2004), and distilled into clear statements aligned with the research questions. The final step was producing the report.

### 4.5 Rigour

Methodological rigour was ensured by addressing the trustworthiness criteria (Nowell et al. 2017). Credibility was supported through purposeful sampling of nurses directly involved in the transition, transparent recruitment reporting and researchers' relevant expertise. The research team acknowledged that their professional roles—particularly the involvement of a Nurse Manager from the same institution—had the potential to influence participants' responses and the interpretation of data. To mitigate this, roles during focus groups were clearly defined to minimise perceived power dynamics, and the Nurse Manager did not lead discussions. In line with interpretive description methodology, member checking was not used, as the focus was on generating broader insights rather than verifying individual accounts. Transferability was supported by detailed descriptions of participants, context and setting. Dependability was achieved via a logical, traceable and documented process. Confirmability was ensured by maintaining an audit trail linking quotes to participants, developing a coding tree and holding regular team discussions to reach consensus on themes.

### 4.6 Ethics

The study was conducted in accordance with the principles outlined in the Declaration of Helsinki (World Medical Association 2024). Full compliance with the General Data Protection Regulation regulations (GDPR Regulation [EU] 2016/679) was ensured, respecting participants' rights regarding their personal data. Privacy and confidentiality were rigorously upheld: all data provided by participants were treated as strictly confidential and anonymity was maintained throughout the research process. Additionally, measures were taken to ensure that participants remained anonymous throughout the study. Results were presented in an anonymised form, and data access was restricted exclusively to the members of the research team.

## 5 Findings

The study included 14 RNs, all of whom were female, with a mean age of 40.8 years. As reported in Table 1, the participants' highest level of education varied: 42.9% (*n* = 6) held a bachelor's degree in nursing, 35.7% (*n* = 5) completed advanced education (e.g., a master's degree), and 21.4% (*n* = 3) held a diploma in nursing. Participants reported an average of 15.8 years of professional nursing experience, with 7.8 years on average of work experience in a respiratory ward.

**TABLE 1:** Characteristics of register nurses.

Characteristics	
Gender, *n* (%)	
Female	14 (100)
Age, years, mean (SD)	40.8 (8.5)
Education, *n* (%)	
Nursing diploma	3 (21.4)
Bachelor's degree	6 (42.9)
Advanced education^a^	5 (35.7)
Working experience, years, mean (SD)	15.8 (8.0)
Working experience in adult respiratory ward, years, mean (SD)	7.8 (5.5)

Abbreviation: SD, standard deviation.

^a^ For example: Master's degree.

The transition experience of skilled respiratory nurses relocating to a newly established IMCU was captured by two interconnected themes: (a) the introspection of waiting amidst change and readiness; and (b) the road to organisational change, marked by both driving forces and obstacles (Table 2). These themes represent complementary perspectives of the transition journey, integrating both the individual psychological processes and the broader systemic dynamics at play.

**TABLE 2:** Coding tree: Themes, categories and codes identified from focus groups.

Themes and categories	Codes	Quotes	Participants
**1. The introspection of waiting amidst change and readiness**			
1.1 Being torn between different facets of change	Feeling enthusiastic about the new professional challenge	‘… When I first found out, I was enthusiastic because, after all, in our ward we have already managed sub‐intensive patients. It seemed stimulating…However, as time went on, I thought about it more and realized that perhaps some fundamental elements are missing. So, yes, the enthusiasm has faded a bit, but ideally, I think it could be a good thing…’. (FG1RN1)	FG1RN1, FG1RN2, FG1RN3, FG2RN3, FG2RN4, FG2RN6
	Experiencing a state of limbo	‘And so, we are still waiting for this relocation… I mean, we're living in a sort of limbo… In the meantime, yes, we see it developing, but…I don't know, it still feels like something… [sentence left incomplete]’. (FG2RN3)	FG1RN2, FG1RN3, FG1RN5, FG1RN6, FG2RN2, FG2RN3, FG2RN4, FG2RN5
	Worrying about patient case mix	‘Yes, because there's this aspect: if all the patients are pulmonology cases, there's experience in that area. However, if patients with other diseases come in, that's when I start questioning myself a bit, especially in critical situations…’. (FG1RN3)	FG1RN1, FG1RN2, FG1RN3, FG1RN5, FG2RN1, FG2RN3, FG2RN2, FG2RN4, FG2RN5
	Worrying about the layout of the new ward	‘The intermediate care unit, if you look at it, you might think it seems adequate, but then add the patients, add the monitors, add the infusion stands, the cabinets for the medications, and everything…To me, it all seems very, very small’. (FG2RN7)	FG1RN2, FG1RN3, FG2RN1, FG2RN4, FG2RN6, FG2RN7
1.2 Perceiving unpreparedness	Feeling inadequate on technical skills	‘Then yes, also technical skills…I mean, if I, as a nurse, had to catheterize an artery right now, I wouldn't know how to do it…Someone would have to teach me’. (FG1RN1)	FG1RN1, FG1RN2, FG1RN3, FG1RN5, FG1RN7, FG2RN2, FG2RN3, FG2RN4, FG2RN5, FG2RN6, FG2RN7
	Fearing mistakes and failures	‘Putting aside all the legal aspects, which I don't even want to think about, but just on a moral level, I can't risk doing something I don't know how to do and therefore making a mistake…I just want to be prepared’. (FG1RN1)	FG1RN1, FG1RN3, FG1RN4, FG1RN5, FG1RN7, FG2RN1, FG2RN5
	Expecting a sudden and disorganised transition	‘Yes, but it will be: ‘Tomorrow you'll do the relocation’ (FG1RN2) ‘And then: “The intermediate care unit opens the day after tomorrow”’. (FG1RN1)	FG1RN1, FG1RN2, FG1RN5, FG1RN6, FG1RN7, FG2RN1, FG2RN3
**2. The road to organisational change with both driving forces and obstacles**			
2.1 Having the right equipment	Advocating for a collective approach	‘But I don't think anyone would step back, some might do it with a bit more fear, others with more enthusiasm…Of course, teamwork is key. We can't think of ourselves as nursing assistants‐nursing assistants, nurses‐nurses, doctors‐doctors, physiotherapists‐physiotherapists…We need to be united; we all need to go in the same direction…’. (FG1RN2)	FG1RN2, FG1RN3, FG1RN5, FG2RN3, FG2RN4, FG2RN5, FG2RN6
	Recognising the need for structured preparation	‘However, in a context where everything is planned, you also need to provide training, and not training that is quick and unstructured, it needs to be something done properly’. (FG2RN2)	FG1RN1, FG1RN2, FG1RN3, FG1RN4, FG1RN5, FG1RN7, FG2RN1, FG2RN2, FG2RN3, FG2RN4, FG2RN6, FG2RN7

Favouring experiential training	‘Well, about training in our workplace, I'm speaking from the heart here because, when the idea of starting to do shifts in other units was first mentioned… In my opinion, a person feels more comfortable and learns better in their own work environment’. (FG2RN2)	FG1RN3, FG1RN5, FG1RN6, FG1RN7, FG2RN1, FG2RN2, FG2RN3, FG2RN4, FG2RN5
2.2 Noticing obstacles along the way	Worrying about physician readiness	‘Honestly, it gave me anxiety, but because, in my opinion, we don't have doctors who are capable of… We don't have doctors, and we also don't have doctors who are capable of managing an intermediate care unit’. (FG1RN7)	FG1RN1, FG1RN2, FG1RN3, FG1RN4, FG1RN5, FG1RN7, FG2RN3, FG2RN4, FG2RN5
	Being undervalued	‘It's true, I didn't feel involved because they never presented us with a project… I mean, there's a lack of sharing the initial project’. (FG2RN3)	FG1RN1, FG1RN2, FG1RN3, FG1RN4, FG1RN5, FG1RN6, FG2RN2, FG2RN3, FG2RN4, FG2RN5, FG2RN6, FG2RN7

Abbreviation: FG1RN1, focus group n.1; registered nurse n.1.

### 5.1 The Introspection of Waiting Amidst Change and Readiness

The initial phase of the transition journey spans from the moment the change is communicated to the nurses' team to the staff's actual relocation. During this waiting period, nurses reflected on the future that lay ahead and their ability to face the upcoming challenges. The anticipation of change resulted in an inner conflict, as they navigated between feelings of excitement and concerns. This theme outlines the introspective and personal dimensions of the transition experience, as reflected in the related categories outlined below.

#### 5.1.1 Being Torn Between Different Facets of Change

Nurses in the early stages of the transition process reported feeling torn between different facets of change. On one hand, there were positive expectations about the future; on the other hand, concerns arose related to the logistical, technical and organisational dimensions of the transition. Initially, some nurses perceived the change as a stimulating opportunity for professional growth and development, particularly drawing from their prior experience in managing semi‐critical patients during the COVID‐19 pandemic. This enthusiasm even prompted one nurse to leave her previous organisation to join the new unit described in the study, motivated by its opening. However, over time, this initial enthusiasm waned, influenced by delays and the perceived lack of cornerstones, understood as essential elements needed to navigate the impending transition:

However, as time went on, I thought about it more and realized that perhaps some fundamental elements are missing. So, yes, the enthusiasm has faded a bit, but ideally, I think it could be a good thing… (FG1RN1)

The transition, initially seen as empowering, becomes destabilising when expectations go unmet. This underscores how professional identity is not only shaped by role and expertise, but also by the presence of institutional trust and clear direction, elements that allow nurses to feel secure, valued, and fully engaged in their evolving roles. The prolonged waiting period and lack of clear communication fostered a sense of suspension and uncertainty, which one nurse described as ‘being in a limbo’:

And so, we are still waiting for this relocation. I mean, we're living in a sort of limbo. In the meantime, yes, we see it developing, but… I don't know, it still feels like something… [sentence left incomplete] (FG2RN3)

The metaphor of ‘limbo’ carries not only an absence of information, but also a disconnection from routine, purpose, and forward momentum—elements central to professional identity and confidence in clinical roles. Nurses also expressed doubts about the patient case mix they would be required to manage in the new setting, citing a lack of sufficient information, along with concerns about their ability to care for patients with conditions unfamiliar or atypical to pulmonary diseases, particularly in critical situations outside their usual area of expertise:

If patients with other diseases come in, that's when I start questioning myself a bit, especially in critical situations… (FG1RN3)

In a high‐acuity environment like an IMCU, where decisions carry weight and rapid responses are essential, unfamiliar case mixes can undermine a nurse's sense of competence and preparedness—core elements of their role. Moreover, the physical layout and limited space of the new IMCU raised concerns about its functionality and ability to support the required workflows:

The sub‐intensive care unit, if you look at it, might seem adequate, but then add the patients, the monitors, the infusion stands… it all seems very, very small (FG2RN7)

A nurse's ability to deliver safe, confident care is tied not just to their skills, but also to how well the environment supports their responsibilities.

#### 5.1.2 Perceiving Unpreparedness

Nurses reported feeling unprepared to face the challenges of the new reality. Participants described a sense of inadequacy regarding their technical skills across several areas (e.g., arterial cannulation) and voiced apprehension about using and operating certain medical devices, such as specific mechanical ventilators, which they anticipated using more frequently in the IMCU but had limited or no prior experience with.

We're not trained, at least speaking for myself, I'm not trained for a sub‐intensive care unit, and I don't think the doctors are either (FG2RN5)

This sense of unpreparedness was compounded by the concern of making mistakes or experiencing failure—perceived as inevitable without sufficient preparation—which emerged as a significant source of anxiety. These concerns were further aggravated by legal and moral implications, as expressed by another participant:

Putting aside all the legal aspects, which I don't even want to think about, but just on a moral level, I can't risk doing something I don't know how to do and therefore making a mistake… I just want to be prepared (FG1RN1)

The nurse framed competence not just as a clinical requirement but as a moral obligation, suggesting that the absence of preparation challenges their fundamental sense of professional integrity. The desire ‘just to be prepared’ is not a request for reassurance, it is a call for institutional accountability to support safe, ethical practice. Several participants also expressed an expectation that the transition to the new IMCU would occur abruptly and with minimal preparation, for example, ‘Tomorrow you'll do the relocation’ (FG1RN2). The anticipated suddenness of the transition evoked feelings of powerlessness and highlighted a lack of control over changes that deeply affect professional roles and responsibilities.

### 5.2 The Road to Organisational Change With Both Driving Forces and Obstacles

In the redeployment journey, organisational factors played a significant role in shaping nurses' adaptation and approach to change. These factors acted as both facilitators and obstacles. This second theme focuses on the external and organisational factors that participants perceived to shape and influence the process.

#### 5.2.1 Having the Right Equipment

Participants identified several factors that could support a smoother redeployment to the new IMCU. Nurses emphasised the importance of teamwork and collaboration across all professional roles, suggesting that a collective approach would foster a more cohesive and effective work environment:

We need to be united; we all need to go in the same direction… Teamwork is key (FG1RN2)

This call for unity reflected more than a functional desire for collaboration; it expressed a deep emotional need for mutual support, psychological safety, and shared responsibility in the face of uncertainty. Additionally, participants stressed the critical need for well‐structured training programmes to build confidence and ensure readiness for the new unit:

In a context where everything is planned, you also need to provide training and not training that is quick and unstructured…it needs to be something done properly (FG2RN2)

Nurses advocated for a comprehensive training approach including specific courses with both theoretical and practical elements, time dedicated to familiarising themselves with the new environment, and mentoring with experts. Participants expressed a clear preference for experiential training delivered within their own clinical environment, emphasising the value of learning through direct engagement in familiar settings:

…In my opinion, a person feels more comfortable and learns better in their own work environment (FG2RN2)

For the participants, learning is most effective not only when it is practical, but when it is situated in an environment that aligns with one's existing routines, relationships, and sense of belonging.

#### 5.2.2 Noticing Obstacles Along the Way

Concerns about medical staffing shortages and the expertise emerged as a recurring theme. Some nurses questioned the team's capacity to effectively manage the IMC;

We don't have physicians, and we also don't have physician who are capable of managing a sub‐intensive care unit (FG1RN7)

Nurses reported the recruitment of inexperienced physicians, such as residents, coinciding with the resignation of senior doctors. This shift led to challenges in adapting to new team members and inefficiencies that increased nursing workload. Nurses feared these difficulties would worsen in the new unit, with fragmented patient care and insufficient collaboration with medical staff further complicating their adaptation. Negative and hesitant attitudes from some physicians, compounded by logistical challenges, were described as significantly undermining the overall work climate. One nurse recalled physicians expressing reluctance to work in the new unit due to staffing shortages:

More than one doctor has told me, “No, I don't want to go there in these conditions, knowing I'd have to cover countless shifts because there isn't enough medical staff” (FG1RN3)

Such perceived negativity was viewed as undermining the broader team morale. Nurses emphasised the importance of fostering a more optimistic and collaborative environment, built on trust and enthusiasm across all professional roles, to establish a supportive and effective work atmosphere:

Because we can't be the ones who stay positive while the doctors are hesitant (FG1RN3)

Another external obstacle identified by experienced nurses was a sense of undervaluation. Nurses expressed dissatisfaction with their limited involvement in the planning and decision‐making processes for the new unit:

I didn't feel involved because they never presented us with a project… There's a lack of sharing the initial project (FG2RN3)

Quotes revealed frustration over the lack of information sharing, exclusion from workspace design, and minimal investment in their training. These experiences undermined their professional identity, making them feel unrecognised and dispensable despite being central to patient care. Participants pointed to a lack of focus on their professional development, particularly in equipping them to meet the challenges of the new IMCU.

## 6 Discussion

This study aimed to explore how nurses experienced the phenomenon of transition into a new IMCU. While IMCUs are well‐established in many regions globally, in some parts of Italy, they represent a relatively new organisational model. This disparity in development can add complexity to resource management and staff distribution, particularly in regions where IMCUs are still in the process of being implemented. The participating nurses expressed two interconnected themes: (a) the introspection of waiting amidst change and readiness and (b) the road to organisational change with both driving forces and obstacles. These themes illustrate the multifaceted nature of work transition experience, encompassing both personal and organisational dimensions. While staff had been deployed to IMCUs during the COVID‐19 pandemic, these assignments occurred under urgent and temporary conditions driven by the emergency context (Danielis et al. 2021). In contrast, the 2024 interviews captured the perspectives of nurses who had been officially and permanently reassigned to the IMCU as part of a planned, long‐term workforce reorganisation. This redeployment, as opposed to an emergency‐driven response, allowed participants to reflect on more stable, structured experiences—offering insights into broader organisational and staffing dynamics beyond the immediate impact of the pandemic.

The first theme captured the emotional and cognitive processes nurses experienced as internal challenges during the initial stages of their transition. Nurses reported a tension between positive emotions, such as enthusiasm for professional growth, and negative expectations, including concerns and uncertainties about the demands of the new role. This emotional ambivalence reflects the nonlinear and dynamic nature of redeployment experienced by nurses, as noted in previous research (Arrowsmith et al. 2016; Chicca and Bindon 2019). It also underscores how uncertainty can emerge early in the transition process, rather than only after entering the new role (Chicca 2021; Clarke et al. 2024). A recent piece of literature highlights that when nurses are redeployed, different patterns of adjustment can emerge (Dunning et al. 2024). These are shaped by varying levels of control, support and contextual understanding, and in some cases, redeployment may threaten professional identity, leading to psychological distress, burnout and increased turnover intentions. The perceived inadequacy, coupled with fears of making mistakes and their potential legal and moral implications, underscores the importance of structured training programmes to alleviate anxiety, build confidence and ensure patient safety (Abdulmohdi and Huang 2025; Chicca 2021; Chicca and Bindon 2019; Dellasega et al. 2009). Moreover, our nurses anticipated a turbulent transition, expressing discomfort with the prospect of being placed into new and demanding clinical settings without sufficient preparation. This perspective aligns with existing literature, which highlights that inadequately supported transitions can heighten stress, diminish professional efficacy, disrupt continuity of care and impede successful adaptation to new roles (Ashley et al. 2018; Chicca 2021; Chicca and Bindon 2019; Fagerdahl et al. 2022). Moreover, resistance to change in nursing is influenced by individual, interpersonal and organisational factors (Cheraghi et al. 2023). Individual nurses' attitudes, often shaped by conflicting emotions, may result in defensive reactions to organisational changes.

The second theme captured the external factors that influenced nurses' ability to adapt during the redeployment process. Among the most significant barriers were concerns about the readiness and competence of medical personnel, particularly physicians, and a widespread perception of being undervalued and excluded from the planning and decision‐making phases. Participants expressed frustration over the lack of communication and transparency, which contributed to a sense of disconnection from the organisational vision. In particular, nurses reported dissatisfaction with their limited participation in the planning of the new IMCU and the lack of initial project sharing by the leadership. This perception of marginal participation increased their uncertainty and doubts, underlining the importance of inclusive planning processes in fostering engagement and minimising resistance. A qualitative study conducted in Australia on the relocation of 31 healthcare professionals across workplace facilities similarly found that minimal involvement in planning was associated with increased dissatisfaction and resistance to change (Clarke et al. 2024). Additionally, shortages in medical personnel and high turnover further exacerbated the difficulties of transition. These challenges were perceived as significant barriers to effective change management, as they disrupted team cohesion, hindered interprofessional collaboration, and negatively impacted overall morale (Pressley and Garside 2023). On the other hand, several facilitators emerged. Many nurses emphasised the importance of a collective, team‐based approach, highlighting the value of interprofessional collaboration to support a successful transition. The need for structured and well‐designed training was consistently mentioned, with participants stressing that training should be practical, thorough, and ideally conducted within their familiar work environment. Experiential learning and on‐the‐job training were preferred over more abstract or externally delivered sessions, as they were perceived to be more effective and less anxiety‐inducing.

These external factors, together with internal emotional responses and expectations, act as key forces that can either support or hinder nurses' transition to new care settings. These dimensions do not operate in isolation; instead, they are deeply interconnected. As noted by Ratnapalan et al. (2024), emotional and organisational factors must be considered jointly, as their interaction significantly influences the success of change implementation (Ratnapalan et al. 2024). Proactive strategies, such as strong leadership, effective communication and targeted education, are critical to managing emotional responses, fostering engagement and reducing resistance to change (Jinga et al. 2024). Furthermore, the key facilitators of successful transitions include cohesive teamwork, structured orientation programmes, preceptorship and mentorship development (Chicca and Bindon 2019; Luk et al. 2025), along with psychologically safe work environments and supportive leadership (Clarke et al. 2024). Opportunities for interdisciplinary meetings, effective nurse‐physician communication and socialisation were also found to enhance team dynamics and integration (Ashley et al. 2018). Moreover, training emerged as a central theme in participant interviews, with a particular value placed on practical, structured and context‐specific learning experiences that facilitate familiarity with the new environment. Nurses expressed a clear preference for training provided within their usual work environments, focusing on its role in building confidence and reducing anxiety. Consistent with the work of Melissant et al. (2024), which critiques the limitations of top‐down interventions for novice nurses, our findings suggest that transition programmes for nurses require a more comprehensive, system‐oriented approach. Such an approach must address the dynamic interaction between individual, team and organisational levels to ensure long‐term integration, team cohesion and improved patient care outcomes. Lastly, the literature reinforces the need for structured, evidence‐based support programmes that are flexible and context‐sensitive (Abdulmohdi and Huang 2025; Chicca and Bindon 2019; Dellasega et al. 2009). Key strategies include preceptorship, mentorship, clinical and interpersonal skills development, situated education and peer knowledge sharing (Luk et al. 2025)—all of which contribute to smoother redeployment and more sustainable workforce development.

### 6.1 Strengths and Limitations of the Work

This study addresses a gap in the literature by examining the role of redeployment of nurses moving into IMCUs. Using the interpretive description methodology, the findings are deeply grounded in participants' experiences, offering practical insights into the complexities of clinical transitions. We collected the data during the initial stages of the redeployment process, ensuring that participants' experiences were captured in a timely manner and closely reflected their immediate perceptions and reactions to the transition. This approach offers an in‐depth understanding of the initial challenges faced by nurses transitioning into an IMCU. The study highlights the adverse effects of limited resources and unclear timelines, emphasising the need for targeted interventions to support successful redeployment.

Despite its strengths, this study has limitations that must be acknowledged. First, the focus was exclusively on nurses; future research should incorporate perspectives from other healthcare professionals, such as nurse assistants, physiotherapists and medical doctors, to provide a more comprehensive analysis of the transition experience. Additionally, gathering insights from a broader range of key stakeholders, such as nurse educators, managers, staff development specialists and preceptors or mentors, could further enrich the understanding of the challenges faced by new‐to‐setting nurses and help inform more effective support strategies. Second, although the findings offer valuable insights, transferability to other settings may be limited due to the specific organisational and clinical characteristics of the IMCU studied. Different institutional cultures, staffing models or national healthcare contexts may influence how redeployment is experienced. Finally, conducting longitudinal research at multiple time points and involving both transitioning nurses and professionals supporting them could yield deeper insights into the evolving dynamics of the redeployment process, ultimately contributing to a more robust understanding of this complex phenomenon.

### 6.2 Implications for Policy and Practice

These findings provide actionable insights for nursing leaders, educators and policymakers developing support strategies for nurses transitioning into new clinical settings. While some of the experiences described by the redeployed nurses—such as enthusiasm for a new professional challenge—are consistent with findings from previous studies on nurse redeployment in general, several aspects appear to be specific to the IMCU context. For example, nurses expressed concern about adapting to the new ward layout and feeling unprepared for the technical demands of the IMCU environment, which required targeted training beyond what is typically needed in general wards. Additionally, some participants voiced apprehension about the readiness of medical staff to manage patients in this intermediate level of care, highlighting the complexity of interprofessional coordination in such units. These contextual factors underscore the unique organisational and clinical challenges associated with IMCUs, distinguishing them from more routine redeployment scenarios.

To inform future redeployment planning, we recommend the development of structured transition pathways tailored to the unique requirements of IMCUs. These should include defined timelines with key milestones (e.g., skills assessments, targeted training, phased onboarding) and mechanisms for early identification of nurses who may require additional preparation. Training programmes should recognise prior experience while addressing individual learning needs and include simulation‐based and in situ experiential training led by critical care experts. Importantly, interprofessional training involving physicians and allied health staff can enhance collaborative readiness, mitigate role confusion and improve overall unit function.

Emotional support mechanisms are equally critical. Nurse leaders should foster psychologically safe environments where staff feel empowered to express uncertainties without fear of judgement. Ongoing debriefings, peer support networks, and access to counselling services can help mitigate psychological distress and reduce burnout during transitions.

At the policy level, findings support the integration of IMCU‐specific training modules and interdisciplinary preparedness standards into workforce planning frameworks. Investing in these areas may promote retention, reinforce professional identity, and improve care quality during organisational transitions.

Lastly, future research should explore the long‐term effects of such initiatives on nurse outcomes and assess how organisational culture and leadership shape the success of redeployment efforts across varying clinical contexts.

## 7 Conclusions

This study highlights the complexity and emotional intensity of transition experiences among experienced nurses redeployed to a newly established IMCU. Effectively addressing both the emotional and practical challenges encountered during this phase is essential for the development of robust support systems. Evidence‐based strategies—including structured training, mentorship and inclusive change management—are critical to enhancing resilience, facilitating adaptation and sustaining high‐quality care in evolving clinical settings. By acknowledging resilience and addressing nurses' specific needs, healthcare leaders can strengthen the workforce and ultimately improve healthcare outcomes.

## Author Contributions

F.M., M.B., M.D.: Made substantial contributions to conception and design, or acquisition of data, or analysis and interpretation of data. S.A., M.V.: Involved in drafting the manuscript or revising it critically for important intellectual content. F.M., M.B., S.A., M.V., M.D.: Given final approval of the version to be published. Each author should have participated sufficiently in the work to take public responsibility for appropriate portions of the content. F.M., M.B., S.A., M.V., M.D.: Agreed to be accountable for all aspects of the work in ensuring that questions related to the accuracy or integrity of any part of the work are appropriately investigated and resolved.

## Ethics Statement

The research project received approval from the Territorial Ethics Committee of the Central‐Eastern Veneto Area (CET) (Prot.0009164/24, internal code CET ANV 2024–28).

## Conflicts of Interest

The authors declare no conflicts of interest.

## Supporting information


**Table S1:** Standards for Reporting Qualitative Research (SRQR).
**Table S2:** Interview guide.

## Data Availability

The data that support the findings of this study are available (in Italian) from the corresponding author upon reasonable request.
