# Supplementary material for: Nurses' Experience of Redeployment to a New Intermediate Care Unit for Respiratory Patients: A Qualitative Study
Source: J Adv Nurs. 2025 Aug 22;82(5):5206–17. doi: 10.1111/jan.70170 (PMC13069199; doi:10.1111/jan.70170)
Supplement: Supplementary file 1 — Table S1: Standards for Reporting Qualitative Research (SRQR). Table S2: Interview guide. [file JAN-82-5206-s001.docx]

**SUPPLEMENTARY TABLE 1 | Standards for Reporting Qualitative Research (SRQR)**

|  |  | **Subheading** |
| --- | --- | --- |
| **Title and abstract** | |  |
|  | Title - Concise description of the nature and topic of the study Identifying the study as qualitative or indicating the approach (e.g., ethnography, grounded theory) or data collection methods (e.g., interview, focus group) is recommended | Title |
|  | Abstract - Summary of key elements of the study using the abstract format of the intended publication; typically includes background, purpose, methods, results, and conclusions | Abstract |
|  |  |  |
| **Introduction** | |  |
|  | Problem formulation - Description and significance of the problem/phenomenon studied; review of relevant theory and empirical work; problem statement | Introduction |
|  | Purpose or research question - Purpose of the study and specific objectives or questions | Introduction |
|  |  |  |
| **Methods** | |  |
|  | Qualitative approach and research paradigm - Qualitative approach (e.g., ethnography, grounded theory, case study, phenomenology, narrative research) and guiding theory if appropriate; identifying the research paradigm (e.g., postpositivist, constructivist/ interpretivist) is also recommended; rationale** | Study design |
|  | Researcher characteristics and reflexivity - Researchers’ characteristics that may influence the research, including personal attributes, qualifications/experience, relationship with participants, assumptions, and/or presuppositions; potential or actual interaction between researchers’ characteristics and the research questions, approach, methods, results, and/or transferability | Data collection |
|  | Context - Setting/site and salient contextual factors; rationale** | Setting and Sample |
|  | Sampling strategy - How and why research participants, documents, or events were selected; criteria for deciding when no further sampling was necessary (e.g., sampling saturation); rationale** | Setting and Sample |
|  | Ethical issues pertaining to human subjects - Documentation of approval by an appropriate ethics review board and participant consent, or explanation for lack thereof; other confidentiality and data security issues | Ethics |
|  | Data collection methods - Types of data collected; details of data collection procedures including (as appropriate) start and stop dates of data collection and analysis, iterative process, triangulation of sources/methods, and modification of procedures in response to evolving study findings; rationale** | Data collection |
|  | Data collection instruments and technologies - Description of instruments (e.g., interview guides, questionnaires) and devices (e.g., audio recorders) used for data collection; if/how the instrument(s) changed over the course of the study | Data collection and Supplementary Table 2 |
|  | Units of study - Number and relevant characteristics of participants, documents, or events included in the study; level of participation (could be reported in results) | Data collection |
|  | Data processing - Methods for processing data prior to and during analysis, including transcription, data entry, data management and security, verification of data integrity, data coding, and anonymization/de-identification of excerpts | Data analysis |
|  | Data analysis - Process by which inferences, themes, etc., were identified and developed, including the researchers involved in data analysis; usually references a specific paradigm or approach; rationale** | Data analysis |
|  | Techniques to enhance trustworthiness - Techniques to enhance trustworthiness and credibility of data analysis (e.g., member checking, audit trail, triangulation); rationale** | Data analysis and Rigor |
|  |  |  |
| **Results/findings** | |  |
|  | Synthesis and interpretation - Main findings (e.g., interpretations, inferences, and themes); might include development of a theory or model, or integration with prior research or theory | Results |
|  | Links to empirical data - Evidence (e.g., quotes, field notes, text excerpts, photographs) to substantiate analytic findings | Results |
|  |  |  |
| **Discussion** | |  |
|  | Integration with prior work, implications, transferability, and contribution(s) to the field - Short summary of main findings; explanation of how findings and conclusions connect to, support, elaborate on, or challenge conclusions of earlier scholarship; discussion of scope of application/generalizability; identification of unique contribution(s) to scholarship in a discipline or field | Discussion |
|  | Limitations - Trustworthiness and limitations of findings | Strengths and limitations |
|  |  |  |
| **Other** | |  |
|  | Conflicts of interest - Potential sources of influence or perceived influence on study conduct and conclusions; how these were managed | Conflict of interest |
|  | Funding - Sources of funding and other support; role of funders in data collection, interpretation, and reporting | Funding sources |

**SUPPLEMENTARY TABLE 2 | Interview guide**

- Introduction with clarification of the study objectives and general rules to follow during the focus group.
- Self-introduction of the researchers.
- Participants are informed about the audio recording of the focus group, the respect for privacy (anonymization of responses), the handling of personal data and the possibility of leaving the discussion at any time.
- Reminder to respect the confidentiality of others' responses.
- Stimulus image as an icebreaker (image of a sub-intensive care unit).
- Questions:

1. *What is the first thing that comes to your mind when you look at this image?*
2. *Could you describe the emotions you felt when you were informed about the transfer to the new sub-intensive care unit?*
3. *How do you feel about the idea of working in this new unit one day?*
4. *How do you perceive your involvement in the opening of a new hospital department? How do you see your role in the creation of this new unit?*
5. *What skills do you think should be developed before transferring to a sub-intensive care setting? In your opinion, what kind of training would be useful?*
6. *What factors do you think could facilitate or hinder the transfer?*
7. *Is there anything else you would like to add?*

- Conclusion of the discussion.
- Summary of the main topics covered and shared.
- Debriefing and thanking the participants.
